# A dynamic and collaborative approach to trial recruitment in safetxt, a UK sexual health randomised controlled trial

**DOI:** 10.1177/17407745221078882

**Published:** 2022-03-05

**Authors:** Lauren Jerome, Kimberley Potter, Ona McCarthy, Melissa Palmer, Megan Knight, Caroline Free

**Affiliations:** 1 London School of Hygiene and Tropical Medicine, London, UK; 2 Unit for Social and Community Psychiatry, Queen Mary University of London, London, UK

**Keywords:** Recruitment, randomised controlled trial, intervention, approach, strategy, sexual health, recruiter, recruiting staff

## Abstract

**Background/Aims:**

Recruiting to target in randomised controlled trials is crucial for providing reliable results, yet many trials struggle to achieve their target sample size. Many trials do not report sufficient, if any, details of their recruitment strategy for others to adapt for their own trials. Furthermore, much of the available evidence describes strategies to improve recruitment aimed at participants, as opposed to strategies aimed at engaging and motivating recruiting staff who are deemed essential for recruitment success. The safetxt trial aimed to recruit 6250 participants, aged 16–24 years, who had either tested positive, or received treatment, for chlamydia/gonorrhoea/non-specific urethritis in the last 2 weeks, from across the United Kingdom into a randomised controlled trial investigating a text message intervention to improve sexual health outcomes. In this article, we describe in detail the recruitment strategies we employed that were primarily aimed at recruiters.

**Methods:**

Recruitment began in April 2016. We built on our recruitment methods established in the pilot trial and developed several strategies to increase recruitment as the trial progressed including optimising site set-up, monitoring recruitment progress and identifying issues, facilitating shared learning, tailored recruitment materials, sustaining motivation, and communication. We describe these strategies in detail and provide practical examples for each.

**Results:**

We combine our strategies for increasing recruitment into one cyclical approach whereby progress is continuously monitored, and interventions to improve recruitment are implemented. The site initiation visits were used to develop a clear recruitment plan and establish good relationships with local site staff. Screening logs were particularly helpful for monitoring recruitment challenges. We facilitated shared learning by organising meetings with recruiting sites and conducting site visits. Tailored recruitment materials helped to promote the trial in clinic environments, and rewards and goals helped sustain motivation among recruiting staff. Finally, at the centre of the approach is good communication which ensured we maintained good relationships with local site staff.

**Conclusion:**

We conducted a large, multi-centre trial and successfully recruited to target. Our dynamic collaborative approach to recruitment described in this paper builds upon previous research by combining suggested good practice into one cyclical approach to recruitment, and providing detailed examples of each strategy. It is not possible to attribute a causal link between our approach and recruitment success overall, or with specific sites or recruiting staff. Nonetheless we describe the processes we used to build a good relationship with recruiting staff and sites, and maintain recruitment of large numbers of participants over the 32 months of the trial. Other researchers can use our approach and adapt our examples for their own trials.

## Background

Despite the importance of achieving the necessary sample size in randomised controlled trials (RCTs), a 2017 review found just 56% of RCTs published in the *Health Technology Assessment* journal between January 2004 and April 2016 recruited to their final recruitment target.^1^ This leaves the remaining trials underpowered and unable to provide reliable evidence, resulting in an ineffective use of funding and potentially beneficial interventions being disregarded through a lack of evidence.^2^ Failure to fully recruit may also waste valuable time and resources, or additional resources may be needed if trials recruit more slowly than expected.^2^

There are a plethora of publications claiming to provide evidence and frameworks for successful recruitment strategies, yet the estimated proportion of trials recruiting to target only improved by 1% between the review in 2017 and an earlier review in 2013.^1^ There are a large number of contextual factors that can influence participant recruitment including site characteristics and capability, topic, and patient population that make trials unique and may make it difficult to generalise strategies from published examples.^3–6^ Furthermore some reports of recruitment strategies such as ‘good engagement with sites’ lack the detail or practical examples required for others to learn from and adapt the strategy for their own RCTs. Peckham et al.^7^ also note the CONSORT guidelines do not specify that recruitment strategy is shared in trial reports, meaning many successfully recruiting trials’ strategies are not reported in a public domain, and other researchers cannot learn from examples of recruitment success. There are numerous studies investigating recruitment strategies targeting participants but very little literature describing strategies targeting recruiters,^2^ despite it being consistently noted that a good relationship with, and the engagement of, recruiting sites is crucial for successful participant recruitment.^7,8^ Uncertainty surrounding how to effectively address recruitment issues remains, and there is considerable scope for improving the sharing and adoption of successful recruitment strategies.

We conducted an RCT investigating a text messaging intervention designed to promote safer sexual health behaviours in young adults in the United Kingdom and successfully recruited to target. Although we cannot claim a direct causal link between our successful recruitment and the specific techniques we used, the aim of this article is to describe the recruitment strategies we developed and implemented throughout the trial. By providing detailed practical examples for each strategy we hope other researchers can adapt and use them in their own trials.

## Methods

The safetxt trial aimed to recruit 6250 participants aged between 16 and 24 years who had either tested positive, or received treatment, for chlamydia/gonorrhoea/non-specific urethritis in the last 2 weeks. Participants also had to own a personal mobile phone, be able to provide informed consent, and were not a known sexual partner of someone already in the trial. Participants were randomly allocated to receive either the intervention text messages, or a control text message once a month, for 12 months. Ethical approval for the trial was provided by The National Research Ethics Service Committee London – Riverside (Reference number 15/LO/1665). A detailed trial protocol has been published elsewhere previously.^9^

We did not pre-specify the number of sites we would open for recruitment, instead sexual health services were opened as recruiting sites if they saw at least five eligible patients per month, and believed they could recruit to at least this target. We originally planned to recruit 5000 participants by April 2018, but due to a lower than expected event rate (sexually transmitted infection positivity) the trial steering committee recommended we increase our sample size and extend recruitment to 6250 participants by December 2018. This recruitment extension was based on our monthly recruitment rate at the time of extending. Following our change in sample size and end date a few of our recruiting sites closed due to having other research commitments, and we opened new sites to replace these. Ultimately, 49 sexual health services across the United Kingdom recruited participants at some point to the trial.

### Initial recruitment strategies

Recruiting sites identified potential participants using different strategies depending on their existing care pathways. This included screening pre-booked appointments, providing eligible patients with trial information at walk-in clinics, reviewing clinic attendance records, and discussing the trial when contacting eligible patients with test results. For those seen in clinic, written informed consent could be obtained in person. Alternatively, eligible patients could complete their consent form online, allowing potential participants to join the study without needing to return to, or extend their time in, clinic. Once consent and baseline measures were obtained, they were entered into the online database, which then allocated the participant to either the intervention or control group.

We presented the trial to recruiting staff at the site initiation visit, provided easy-to-read eye-catching patient information leaflets (Supplemental material – Appendix 1), and provided patient facing posters (Supplemental material – Appendix 2) for clinic areas. We encouraged sites to have a computer in clinic that could be used by participants to enrol online and to also follow up with potential participants who were interested in the trial but had not enrolled after a few days.

### Additional recruitment strategies developed

Recruiting such a large number of participants proved challenging. Moreover, as the trial progressed public funding to sexual health services was reduced and many services reorganised,^10^ meaning we had to revisit and update our recruitment approach to work within new service configurations. To address recruitment challenges, we optimised the initial site set-up and developed four techniques to facilitate and improve recruitment.

#### Initial site set-up

Site initiations were conducted with every site prior to recruitment starting. Clinic staff planned how they would identify and approach eligible patients taking into consideration their local care pathways. We also discussed possible recruitment barriers within their clinic and how these could be overcome. Recruiting staff had a range of roles including health advisers, research assistants, and research nurses. The composition of the team varied between clinics, and each site had a ‘site lead’ who was our primary point of contact. We found it most productive if recruitment strategies included all clinic staff, not just those actively recruiting, as this helped to develop a recruitment strategy considering all points in their care pathway, and all staff could engage with identifying potential participants. Therefore, we asked sites to have all staff present at the site initiation and we attended the training in-person wherever possible to promote engagement with all clinic staff.

#### Technique 1: monitoring recruitment progress and identifying issues

This involved continually evaluating recruitment progress throughout the recruitment period by monitoring recruitment and declination numbers for each site. When sites experienced periods of slow recruitment compared to their usual rate, or below their originally expected recruitment rate, we discussed potential issues with the recruiting teams and came up with a solution together. The interventions described in Techniques 2 to 4 were used to address identified recruitment issues.

Another method for monitoring recruitment progress was reviewing the screening log data collected from sites on a monthly basis, allowing us to identify and address systemic recruitment challenges. The screening logs highlighted any sites experiencing a high rate of declinations suggesting we revisit their recruitment strategy. They also included any reasons given by patients for declining to participate (see Supplemental material – Appendix 3). Commonly occurring reasons were discussed by the trial management team and possible solutions fed back to sites.

#### Technique 2: facilitating shared learning

We facilitated shared learning of recruitment skills and strategies for increasing recruitment, primarily by encouraging collaboration between sites. We held four teleconferences throughout the trial, with the main focus being to identify and discuss common recruitment challenges and share ideas for how to overcome them. Recruiting staff also shared ideas for motivating their teams and how they had increased recruitment at their site. Groups for the teleconferences were split between 2 days, and we ensured there were both high and low recruiting sites in each so as to best facilitate mutual learning. We disseminated the discussion points with all sites after the meetings. Although the discussions were organised by us and centred on recruitment, we encouraged the sites to lead the discussions, which enabled them to take ownership of their ideas and successes. We also held two face-to-face meetings in London for all sites to attend. Nurses from three sites gave presentations on their experiences recruiting, and we had talks from speakers on topics related to recruiting in clinical trials, as well as a workshop on effective communication.

Another approach we took to facilitate shared learning of recruitment skills was revisiting our recruiting sites. By physically visiting sites who recruited well and those that were not recruiting so well helped to highlight which practices were enabling recruitment that could be adopted by low recruiting sites, for example, the placement of trial posters or reminders in clinic areas. Visiting sites after there had been staff changes, and asking all clinic staff to be available, was designed to ensure new staff were brought up to speed on best recruitment practices and refresh engagement of all staff with the trial.

#### Technique 3: tailored recruitment materials

We developed recruitment supporting materials including posters aimed at potential participants, posters aimed at clinic staff, stickers for computer monitors, and checklists for clinicians to make note of any eligible patients (see Supplemental material – Appendix 4). Recruitment packs of these materials were sent to all sites and feedback was encouraged. We also encouraged sites to share any locally produced materials for promoting recruitment. For example, reminder stickers and computer pop-ups were shared and adapted for other sites to use locally. To address new challenges, refresh information, and incorporate feedback, materials designed to improve recruitment were continually updated as recruitment progressed.

#### Technique 4: sustaining motivation

We used a number of methods designed to help increase and sustain motivation in our final intervention to increase recruitment.

*Achievable goals*. One method was to provide individually tailored targets and feedback for each recruiting site. The trial management team, in discussion with local recruiting staff, set achievable and realistic targets each month, for example, one participant more than the previous month. Monthly targets would take into consideration the site’s original recruitment target and recruitment rates in previous months to ensure targets provided some challenge but were achievable. The rationale for this was when sites are able to meet their target this increases their confidence and motivation to recruit again.

*Feedback and rewards*. We also motivated sites by offering a number of conditional and unconditional rewards. Sites who met or exceeded targets were highlighted in our monthly newsletters and e-bulletins that were distributed to everyone involved in the trial, and sites also received individual feedback from the trial management team. This was by phone call or email, praising their recruitment efforts and highlighting how important their achievement was to the overall success of the trial.

We held frequent competitions that were not always contingent on recruiting the highest numbers to win. For example, every site who recruits one more participant than last month is entered into a prize draw, with three hampers of snacks and branded tokens, such as mugs, to be won (see Supplemental material – Appendix 5).

We sent unconditional incentives during periods of slow recruitment, such as holiday periods, to all recruiting sites. This included motivational recruitment packs containing recruitment materials along with sweets or biscuits. We sent letters to the sites’ Chief Executives, thanking the research team for their efforts in the trial (see Supplemental material – Appendix 6). Finally, we provided awards for reaching milestones. This was either an award to the site generally, or certificates were provided to specific staff who had recruited a certain number of participants or were nominated by their site lead as a top recruiter (see Supplemental material – Appendix 7). These awards could be included in their appraisals.

#### Communication

Central to our approach was effective communication and regular contact. Good communication has been described as a process that considers the hearer rather than just transferring information. It is also important to build a common understanding, be clear and respectful, and recognise the limitations of emails.^11^ With this in mind, building a good relationship with sites was important to have effective communication, and we used a range of communication methods for sharing different types of information. We conducted visits and used telephone, e-mail, letters, newsletters, social media, and our website to convey information to our sites and to check in on a regular, often weekly, basis. Regular contact aimed to develop these good relationships, keep the trial in the forefront of the sites’ minds, and enable us to be aware of any possible issues as, or even before, they arise. We responded to queries quickly to show sites their input is valued. Moreover, keeping in regular contact with site leads allowed us to monitor the workload of our recruiting sites. If there were competing studies that required attention, having a good relationship with sites meant we were aware of deadlines and could try to adapt recruitment strategies.

*Newsletters*. Newsletters were our primary form of regular communication with sites. At the start of every month during the recruitment period, we issued a newsletter that was sent to all clinic staff. We tried to obtain emails from all clinic staff at the site initiation, and encouraged site leads to forward on newsletters locally. The newsletters covered a range of topics and presented information in a variety of ways to make sure they were interesting and relevant.

Recruitment updates were shared in every newsletter. Overall recruitment numbers were always presented, with different additional monthly figures highlighting a variety of other recruitment numbers every month. This could include sites whose monthly figure had improved, top recruiting leader boards, and sites who reached recruitment milestones – such as recruiting 100 participants.

We also included staff profiles of the trial management team in the initial newsletters and whenever staff joined the team. These profiles briefly highlighted the staff member’s role on the trial, a ‘fun fact’ and contact details. This was designed to help to build relationships with the sites, provide points of contact, and encourage them to contact us.

The newsletters included site updates such as new sites opening and interviews with recruiters that shared recruitment processes and tips. Other updates the newsletters highlighted were the introduction of certificates for staff who recruited participants, competitions, and staff who had been nominated as a top recruiter. Wherever possible we included pictures of clinic staff to help build a sense of community among all our recruiting sites.

Finally, the newsletters were a useful space for communicating any general trial updates and information. This included key changes from amendments, any upcoming conferences or meetings the trial was being presented at or we were hosting, reminders of the eligibility criteria, and how to use the enrolment website. We also used the newsletters in a visually appealing way to highlight feedback from the meetings we held for the clinic staff, rather than simply listing feedback in a word document, and to highlight participant concerns from the screening logs and provide solutions for addressing these. We used the newsletters to provide updates on other aspects of the trial that were ongoing, such as how follow-up was progressing, including positive quotes from participant feedback (see Supplemental material – Appendix 8 for examples of newsletters). See Table 1 for a summary of the techniques developed to facilitate and improve recruitment.

**Table 1.** Summary of techniques.

Technique	Key points
Technique 1. Monitoring recruitment progress and identifying issues	•Monitor recruitment and declination numbers throughout the trial •Discuss dips in recruitment with recruiting staff •Review screening log data and address reported challenges
Technique 2. Facilitating shared learning	•Teleconferences with recruiting staff •Investigator meetings with all sites •Visit recruiting sites and share good practices
Technique 3. Tailored recruitment materials	•Develop recruitment supporting materials e.g. posters, checklists, stickers •Share locally produced materials among all sites •Update materials as the trial progresses
Technique 4. Sustaining motivation	•Individual tailored, realistic targets •Individual feedback and highlight successes •Recruitment competitions, not always contingent on recruiting the ‘most’ •Unconditional incentives during periods of slow recruitment •Letters to Chief Executives •Awards for milestones that can be included in appraisals

## Results

**Figure 1.**
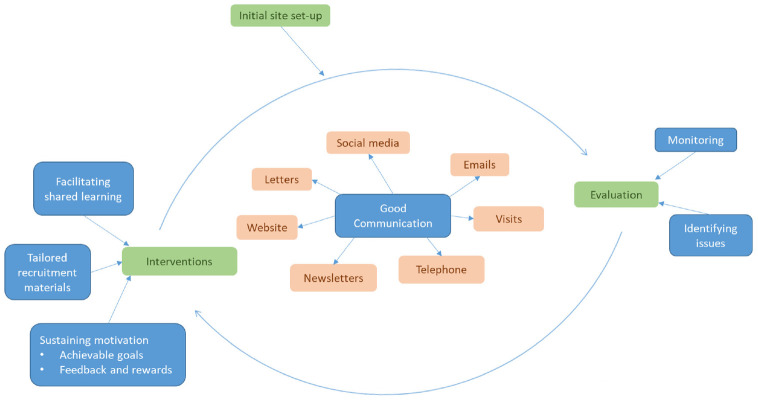
Cyclical approach to recruitment diagram.

The processes we described can be summarised as a cyclical approach to trial recruitment, which at its core was collaborative involving strong communication and also dynamic, adapting to new challenges over time. Within this, Technique 1 (Monitoring recruitment progress and identifying issues) focussed on improving how we monitored and evaluated our recruitment progress, while Techniques 2 to 4 (Facilitating shared learning, Tailored recruitment materials and Sustaining motivation) provided interventions designed to increase recruitment numbers. After new approaches were implemented, Technique 1 helped us to continue to evaluate recruitment progress, resulting in our continuous cyclical approach to supporting recruitment. Communication was central to our approach allowing us to effectively monitor recruitment, respond to challenges, and engage with recruiting sites (Figure 1).

Based on the recruitment strategies discussed at the site initiations, 88% of clinics screened pre-booked appointments and 60% of clinics identified patients from walk-in appointments. The strategy used depended on the type of appointments the clinic had, with many clinics having both types of appointments available; 69% of clinics planned to recruit patients when contacting them either by phone or text message with their test results. The clinics who did not use this approach did not do so for a number of reasons, including not being able to edit outgoing text messages, and a lack of Good Clinical Practice training among the relevant staff. Although local recruitment strategies were planned at the site initiation, over the recruitment period there were changes made to many sexual health services, and so the recruitment strategies used within clinics changed over the course of the trial.

The initial site set-up was crucial to successfully plan a recruitment strategy that considered local contexts and potential recruitment barriers prior to recruitment starting. We used these meetings to establish positive relationships with the sites, which enabled good communication and encouraged regular updates. We were able to have a productive collaborative approach to recruitment as the trial progressed based on the relationships we established at these initial meetings.

Throughout the trial screening logs in particular proved a valuable resource for monitoring and addressing recruitment challenges. Providing solutions to common reasons for refusal seemed to reassure patients, and also increase recruiting staffs’ confidence in the discussions they have with potential participants. For example, after reviewing recorded reasons for refusal, 5% (41/834) of patients had said they were concerned about receiving post at their home address. We sent recruiting staff a mock version of the follow-up envelopes to show patients when they discussed the trial. The aim of this was to reassure patients that the letters would be recognisable and did not include any identifiable markings that would disclose where or who they were sent from.

Facilitating shared learning by organising meetings and conducting site visits was important as it provided opportunities for sites to collaborate and learn from each other’s experiences, generated practical ways to improve recruitment, helped local staff to optimise their clinic environment for recruitment, and increased staff confidence in their recruiting skills.

Tailored recruitment materials helped to promote the trial in clinic environments, and updating these materials regularly meant they remained relevant and eye-catching. Sharing materials between sites was beneficial for staff who work in similar clinic environments, where the trial management team may not always have the best insight for how to highlight recruiting in their day-to-day practice. This also motivated the sites that produced the materials, who we named and thanked in our newsletters.

Rewards to sites, both conditional and unconditional, were crucial to motivate sites to continue putting efforts into recruiting. Setting achievable goals was used to help to motivate sites who may lack experience and resources, or see fewer patients, while still pushing them a little bit further.

We considered good communication as aiding all of our techniques and enabling us to recruit successfully. Our newsletters were designed to keep sites engaged with the trial. Including participant quotes in the newsletters meant sites could see that participants felt positively about the trial. We hoped this would encourage sites to approach patients, help recruiting staff see the participant perspective, and show that trial participation contributed to mutual goals of improving the sexual health of young people.

## Discussion

We employed a dynamic, collaborative, cyclical approach to recruitment and successfully recruited to target in our large, multi-centre RCT. In our approach to recruitment, progress is monitored and reviewed, interventions designed to address recruitment challenges are implemented, and recruitment progress following these interventions continues to be monitored and reviewed. The initial site set-up builds a foundation for successful recruitment. By building a strong relationship with recruiting sites recruitment barriers can be identified and solutions can be created. At the core of the approach is regular, engaging, responsive communication, which we see as key to successful recruitment, and without which we believe intervention attempts to improve recruitment will have limited success. Throughout the trial we used four techniques, one to monitor progress, and three provided interventions to respond to identified issues. We recruited 6250 young people into a trial regarding the sensitive topic of sexual health, on time over 32 months. Although we cannot attribute causality to one or any specific element(s) of the processes we used, we consider our overall co-ordinated approach was important in helping us recruit a large number of participants on time.

Previous research has demonstrated the effectiveness of some elements of our approach to recruitment. Presenting and planning at, and even just having, a site initiation has been suggested to be beneficial for trial recruitment.^2^ We have expanded upon this by describing how the site set-up can be optimised. Regular and personalised feedback is consistently reported as key for recruitment success.^2,4,8,12,13^ Good communication, including regular and personal feedback, has been described and is central in our approach. Good communication enables collaboration with sites on recruitment strategy and facilitates a reactive approach to tackling recruitment issues. Individual feedback is important for demonstrating to each and every site their importance in the trial, and to increase their self-efficacy in recruiting. We provide detailed examples of how newsletters in particular can be used to aid communication. Constant, real-time monitoring has been suggested to contribute to optimal trial performance.^4,14,15^ We have described monitoring in our approach, as well as how screening logs can be used effectively for this purpose. Site visits and sharing recruiter experiences, recruitment materials, and incentives have also been suggested to be beneficial for recruitment.^4,12,16,17^ We build upon these by describing a number of ways to facilitate shared learning, tailor recruitment materials, and sustain motivation. Finally, we have brought all these elements together in one cyclical approach to recruitment.

Other recruitment models, such as the recruitment optimisation model,^14^ also describe optimising recruitment as a dynamic cycle, rather than a linear process. However, their model primarily focuses on how marketing strategies can be utilised to devise segment-specific recruitment strategies targeting patients directly. Our approach focuses on strategies targeting recruiters and provides examples from each step of the cycle that could be adapted for other trials.

The primary limitation of our approach is that we did not test our techniques in such a way that cause and effect could be established. We recognise the limitations of this, in that we cannot confidently say which, if any, aspects of our approach to recruitment were most important in recruiting to target. However, Walters et al.^1^ 2017 review found just 56% of trials met their recruitment target, and multi-centre trials achieved an average recruitment rate of 0.86 patients per centre per month. We met our recruitment target and exceeded this average recruitment rate. Therefore, we believe our overall approach to recruitment played a role in helping us to recruit successfully.

Specific trial settings and participant populations may be important in themselves for how well recruitment interventions work. Within our trial many services were reorganised during the recruitment period,^10^ meaning our setting changed and our approach to recruitment had to be revisited and adapted. Moreover, our trial investigated a relatively non-invasive intervention that was not time-consuming for the participant. Trials with more complex interventions and procedures may require more complex techniques for improving recruitment. However, as our approach is aimed at engaging and motivating recruiting staff, we believe it can be beneficial in most settings. In this article, we aimed to provide sufficient detail and examples for researchers to assess which elements of our approach might be transferable to their own RCT, or which parts can be adapted to fit other contexts.

## Conclusion

Our dynamic collaborative approach to recruitment describes activities deemed important for recruitment success in clinical trials in one cyclical approach, involving constant evaluation and development of new approaches to overcome recruitment challenges. We hope our approach described, and examples given, will be of use to other researchers conducting RCTs.

## Supplemental Material

sj-docx-1-ctj-10.1177_17407745221078882 – Supplemental material for A dynamic and collaborative approach to trial recruitment in safetxt, a UK sexual health randomised controlled trial Click here for additional data file. Supplemental material, sj-docx-1-ctj-10.1177_17407745221078882 for A dynamic and collaborative approach to trial recruitment in safetxt, a UK sexual health randomised controlled trial by Lauren Jerome, Kimberley Potter, Ona McCarthy, Melissa Palmer, Megan Knight and Caroline Free in Clinical Trials

sj-docx-2-ctj-10.1177_17407745221078882 – Supplemental material for A dynamic and collaborative approach to trial recruitment in safetxt, a UK sexual health randomised controlled trial Click here for additional data file. Supplemental material, sj-docx-2-ctj-10.1177_17407745221078882 for A dynamic and collaborative approach to trial recruitment in safetxt, a UK sexual health randomised controlled trial by Lauren Jerome, Kimberley Potter, Ona McCarthy, Melissa Palmer, Megan Knight and Caroline Free in Clinical Trials

sj-docx-3-ctj-10.1177_17407745221078882 – Supplemental material for A dynamic and collaborative approach to trial recruitment in safetxt, a UK sexual health randomised controlled trial Click here for additional data file. Supplemental material, sj-docx-3-ctj-10.1177_17407745221078882 for A dynamic and collaborative approach to trial recruitment in safetxt, a UK sexual health randomised controlled trial by Lauren Jerome, Kimberley Potter, Ona McCarthy, Melissa Palmer, Megan Knight and Caroline Free in Clinical Trials

sj-docx-4-ctj-10.1177_17407745221078882 – Supplemental material for A dynamic and collaborative approach to trial recruitment in safetxt, a UK sexual health randomised controlled trial Click here for additional data file. Supplemental material, sj-docx-4-ctj-10.1177_17407745221078882 for A dynamic and collaborative approach to trial recruitment in safetxt, a UK sexual health randomised controlled trial by Lauren Jerome, Kimberley Potter, Ona McCarthy, Melissa Palmer, Megan Knight and Caroline Free in Clinical Trials

sj-docx-5-ctj-10.1177_17407745221078882 – Supplemental material for A dynamic and collaborative approach to trial recruitment in safetxt, a UK sexual health randomised controlled trial Click here for additional data file. Supplemental material, sj-docx-5-ctj-10.1177_17407745221078882 for A dynamic and collaborative approach to trial recruitment in safetxt, a UK sexual health randomised controlled trial by Lauren Jerome, Kimberley Potter, Ona McCarthy, Melissa Palmer, Megan Knight and Caroline Free in Clinical Trials

sj-docx-6-ctj-10.1177_17407745221078882 – Supplemental material for A dynamic and collaborative approach to trial recruitment in safetxt, a UK sexual health randomised controlled trial Click here for additional data file. Supplemental material, sj-docx-6-ctj-10.1177_17407745221078882 for A dynamic and collaborative approach to trial recruitment in safetxt, a UK sexual health randomised controlled trial by Lauren Jerome, Kimberley Potter, Ona McCarthy, Melissa Palmer, Megan Knight and Caroline Free in Clinical Trials

sj-docx-7-ctj-10.1177_17407745221078882 – Supplemental material for A dynamic and collaborative approach to trial recruitment in safetxt, a UK sexual health randomised controlled trial Click here for additional data file. Supplemental material, sj-docx-7-ctj-10.1177_17407745221078882 for A dynamic and collaborative approach to trial recruitment in safetxt, a UK sexual health randomised controlled trial by Lauren Jerome, Kimberley Potter, Ona McCarthy, Melissa Palmer, Megan Knight and Caroline Free in Clinical Trials

sj-docx-8-ctj-10.1177_17407745221078882 – Supplemental material for A dynamic and collaborative approach to trial recruitment in safetxt, a UK sexual health randomised controlled trial Click here for additional data file. Supplemental material, sj-docx-8-ctj-10.1177_17407745221078882 for A dynamic and collaborative approach to trial recruitment in safetxt, a UK sexual health randomised controlled trial by Lauren Jerome, Kimberley Potter, Ona McCarthy, Melissa Palmer, Megan Knight and Caroline Free in Clinical Trials

## References

[1] Walters SJ Bonacho dos Anjos Henriques-Cadby I Bortolami O , et al. Recruitment and retention of participants in randomised controlled trials: a review of trials funded and published by the United Kingdom Health Technology Assessment Programme. BMJ Open 2017; 7: e015276. 10.1136/bmjopen-2016-015276 PMC5372123 28320800

[2] Treweek S Pitkethly M Cook J , et al. Strategies to improve recruitment to randomised trials. Cochrane Database Syst Rev 2018; 2(2): MR000013. 10.1002/14651858.MR000013.pub6 PMC7078793 29468635

[3] Iribarren SJ Ghazzawi A Sheinfil AZ , et al. Mixed-method evaluation of social media-based tools and traditional strategies to recruit high-risk and hard-to-reach populations into an HIV Prevention Intervention Study. AIDS Behav 2018; 22(1): 347–357. 29124420 10.1007/s10461-017-1956-6 PMC5760436

[4] Farrell B Kenyon S Shakur H . Managing clinical trials. Trials 2010; 11: 78. 20626885 10.1186/1745-6215-11-78 PMC2917433

[5] Kalpakidou AK Cape J Limbachya TJ , et al. Barriers to recruitment when conducting a commissioned randomised controlled trial of medication versus psychological therapy for generalised anxiety disorder: some lessons learned. Trials 2019; 20(1): 284. 31126337 10.1186/s13063-019-3385-5 PMC6534845

[6] McMullen H Griffiths C Leber W , et al. Explaining high and low performers in complex intervention trials: a new model based on diffusion of innovations theory. Trials 2015; 16: 242. 26026849 10.1186/s13063-015-0755-5 PMC4465492

[7] Peckham E Arundel C Bailey D , et al. Successful recruitment to trials: findings from the SCIMITAR+ Trial. Trials 2018; 19(1): 53. 29351792 10.1186/s13063-018-2460-7 PMC5775553

[8] Isaksson E Wester P Laska AC , et al. Identifying important barriers to recruitment of patients in randomised clinical studies using a questionnaire for study personnel. Trials 2019; 20(1): 618. 31666093 10.1186/s13063-019-3737-1 PMC6822437

[9] Free C McCarthy OL Palmer MJ , et al. Safetxt: a safer sex intervention delivered by mobile phone messaging on sexually transmitted infections (STI) among young people in the UK – protocol for a randomised controlled trial. BMJ Open 2020; 10(3): e031635. 10.1136/bmjopen-2019-031635 PMC7064138 32152156

[10] Robertson R . Sexual health services and the importance of prevention. London: The King’s Fund, 2018.

[11] Jarvenpaa SL Keating E . When do good communication models fail in global virtual teams? Org Dyn 2021; 50: 100843.

[12] Dickson S Logan J Hagen S , et al. Reflecting on the methodological challenges of recruiting to a United Kingdom-wide, multi-centre, randomised controlled trial in gynaecology outpatient settings. Trials 2013; 14: 389. 24228935 10.1186/1745-6215-14-389 PMC3835655

[13] Huang GD Bull J Johnston McKee K , et al. Clinical trials recruitment planning: a proposed framework from the clinical trials transformation initiative. Contemp Clin Trials 2018; 66: 74–79. 29330082 10.1016/j.cct.2018.01.003

[14] Galli L Knight R Robertson S , et al. Using marketing theory to inform strategies for recruitment: a recruitment optimisation model and the txt2stop experience. Trials 2014; 15: 182. 24886627 10.1186/1745-6215-15-182 PMC4057570

[15] Menon U Gentry-Maharaj A Ryan A , et al. Recruitment to multicentre trials: lessons from UKCTOCS: descriptive study. BMJ 2008; 337: a2079. 19008269 10.1136/bmj.a2079 PMC2583394

[16] Mitchell EJ Godolphin PJ Meakin G , et al. Do investigator meetings improve recruitment rates in clinical trials? A retrospective before-and-after study of data from nine multi-centre clinical trials. Trials 2020; 21(1): 514. 32522228 10.1186/s13063-020-04465-1 PMC7288550

[17] Smith V Clarke M Begley C , et al. SWAT-1: the effectiveness of a ‘site visit’ intervention on recruitment rates in a multi-centre randomised trial. Trials 2015; 16: 211. 25958221 10.1186/s13063-015-0732-z PMC4429599

