# Supplementary figures and images for: A dynamic and collaborative approach to trial recruitment in safetxt, a UK sexual health randomised controlled trial
Source: Clin Trials. 2022 Mar 5;19(3):251–8. doi: 10.1177/17407745221078882 (PMC9203664; doi:10.1177/17407745221078882)

## Appendix 1. Patient Information Leaflet

*Front*


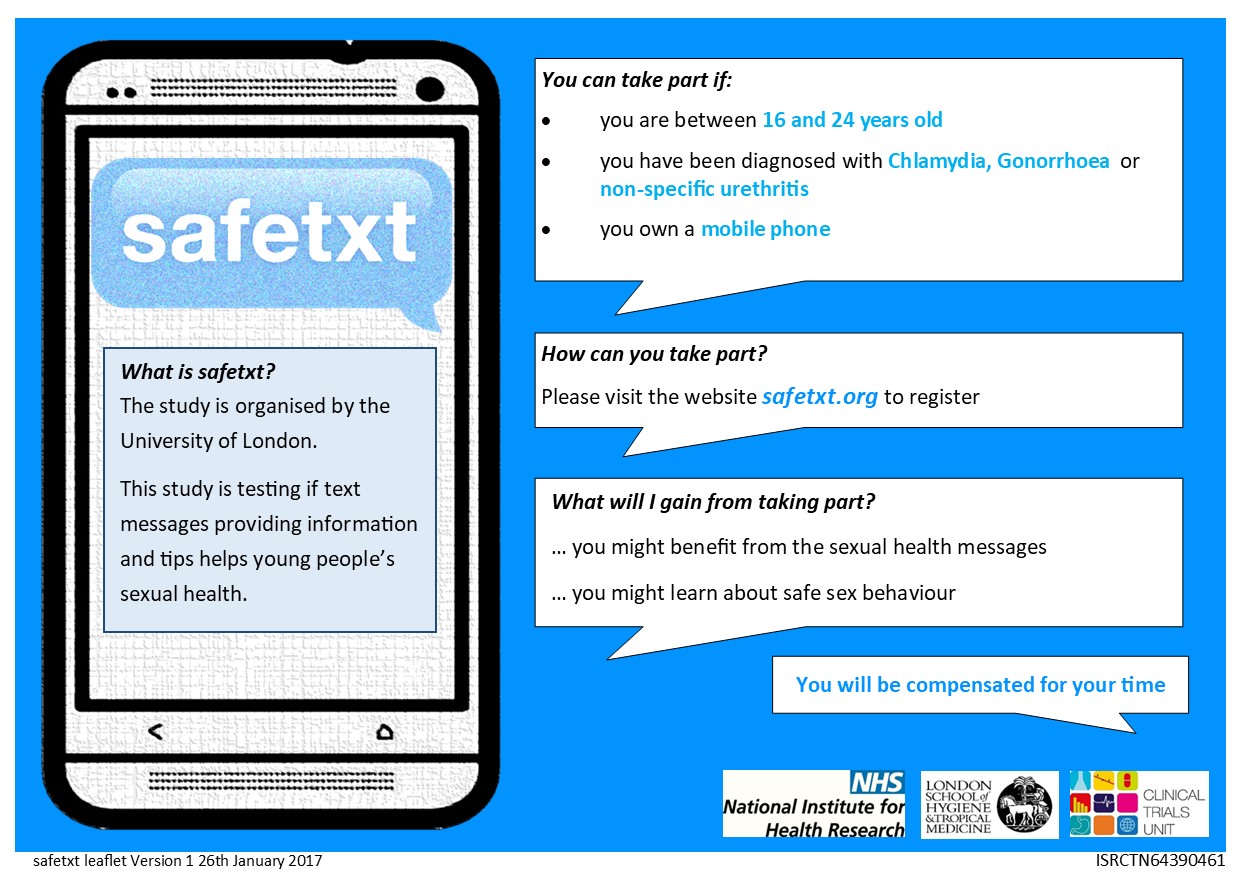


*Back*


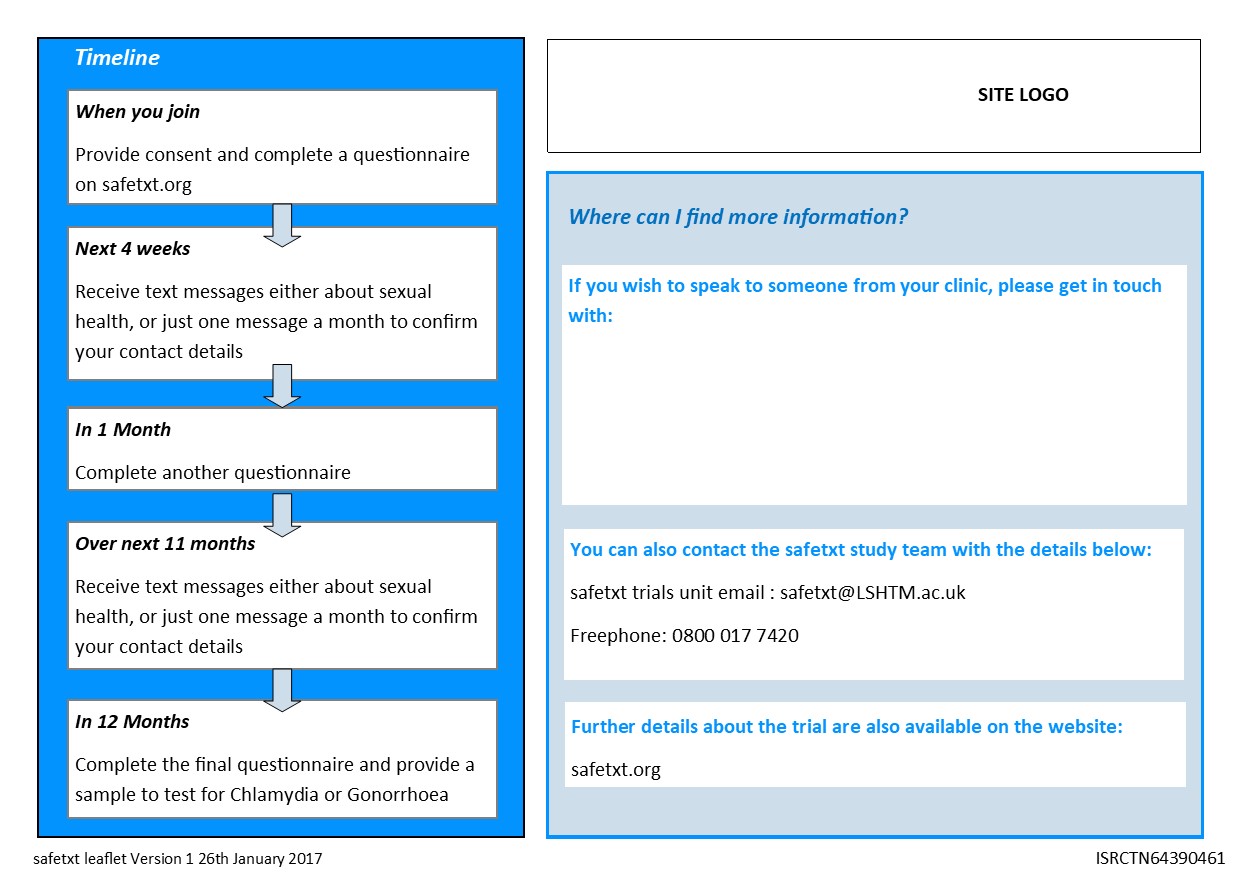

Supplement: sj-docx-1-ctj-10.1177_17407745221078882 – Supplemental material for A dynamic and collaborative approach to trial recruitment in safetxt, a UK sexual health randomised controlled trial [file sj-docx-1-ctj-10.1177_17407745221078882.docx]

## Appendix 2. Patient facing posters

##
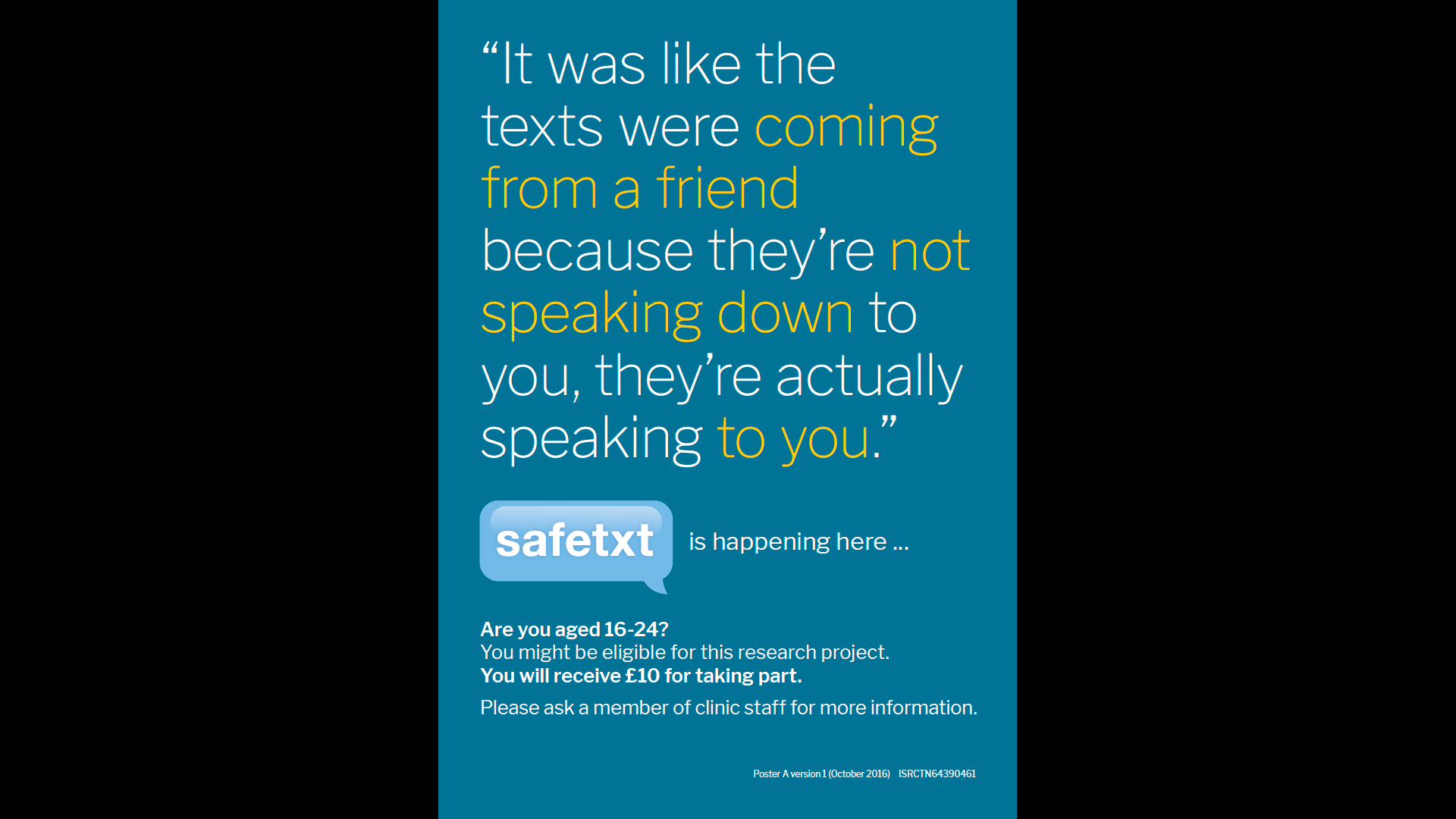


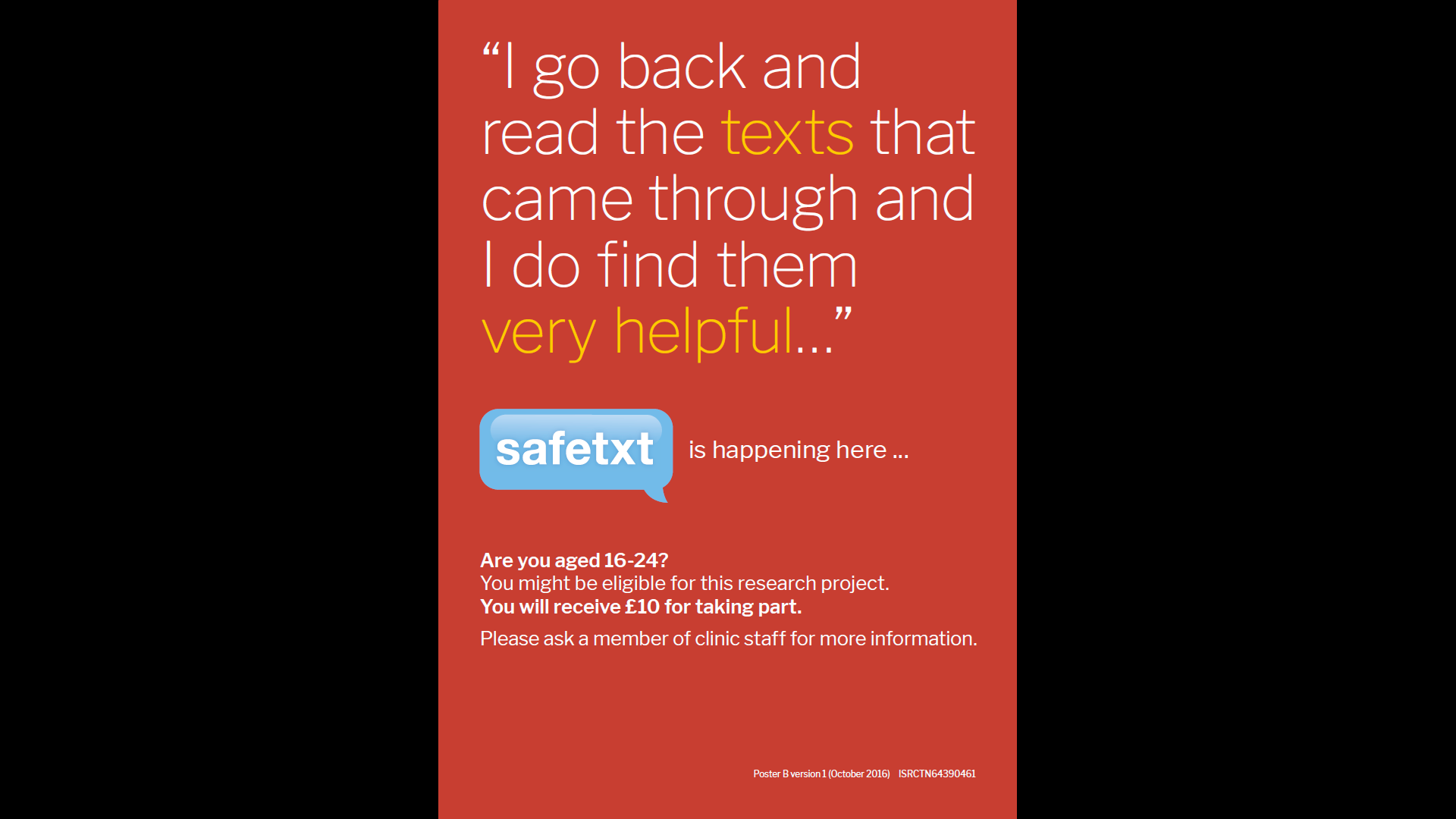


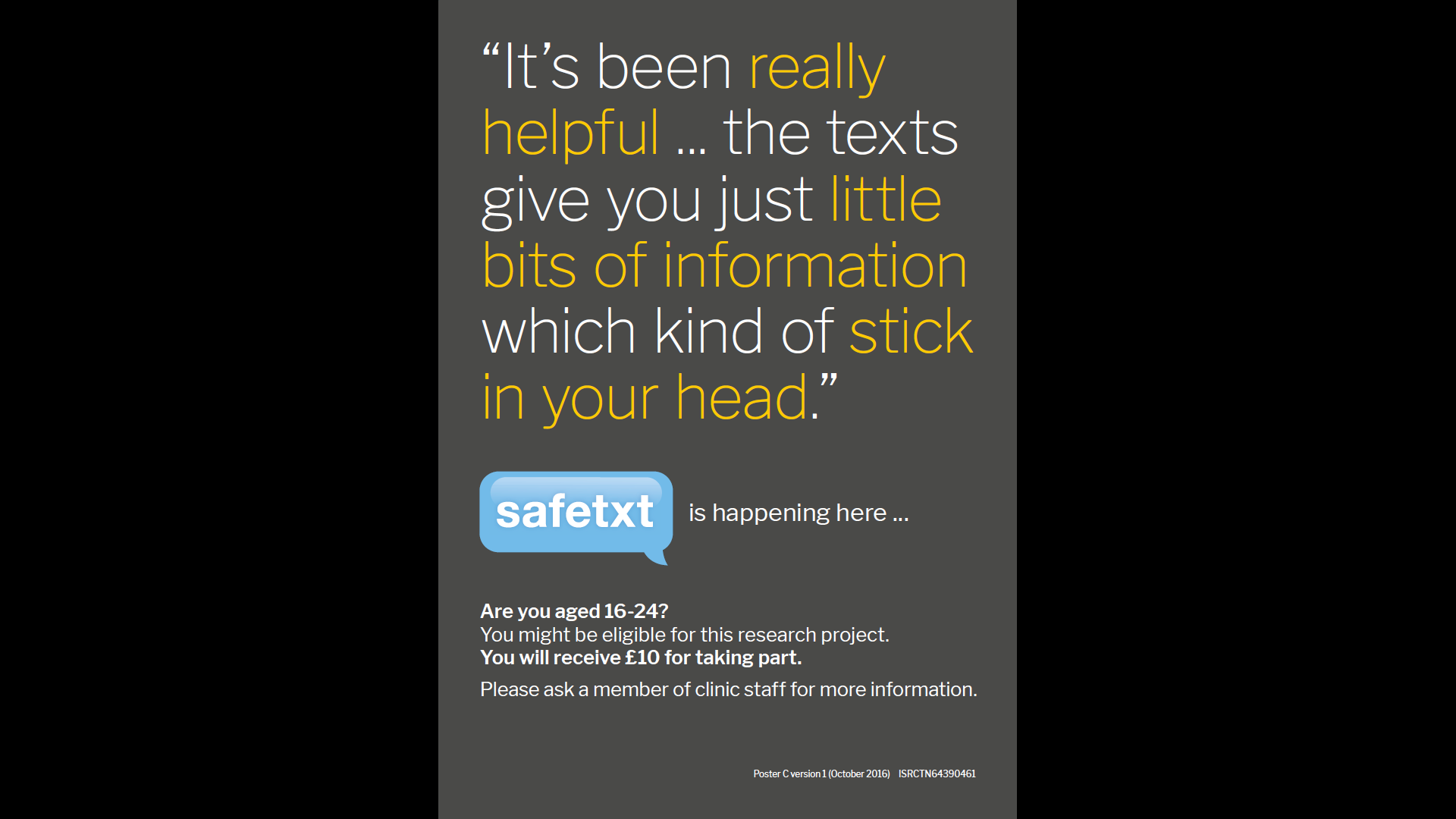

Supplement: sj-docx-2-ctj-10.1177_17407745221078882 – Supplemental material for A dynamic and collaborative approach to trial recruitment in safetxt, a UK sexual health randomised controlled trial [file sj-docx-2-ctj-10.1177_17407745221078882.docx]

## Appendix 5. Hamper prize for one of our competitions.


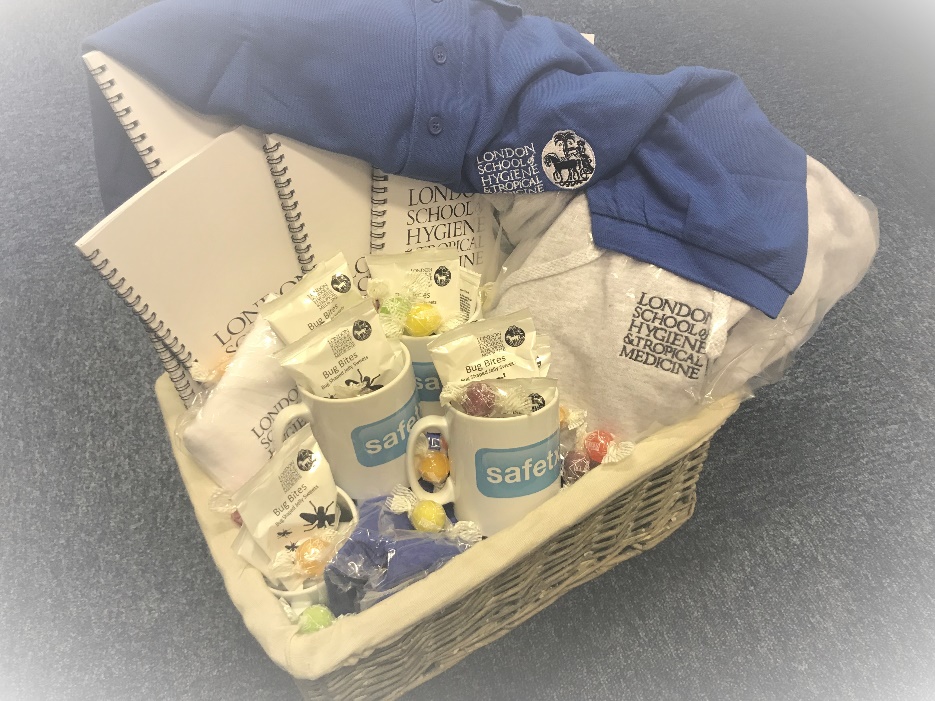

Supplement: sj-docx-5-ctj-10.1177_17407745221078882 – Supplemental material for A dynamic and collaborative approach to trial recruitment in safetxt, a UK sexual health randomised controlled trial [file sj-docx-5-ctj-10.1177_17407745221078882.docx]

## Appendix 6. Letter to a Chief Executive.


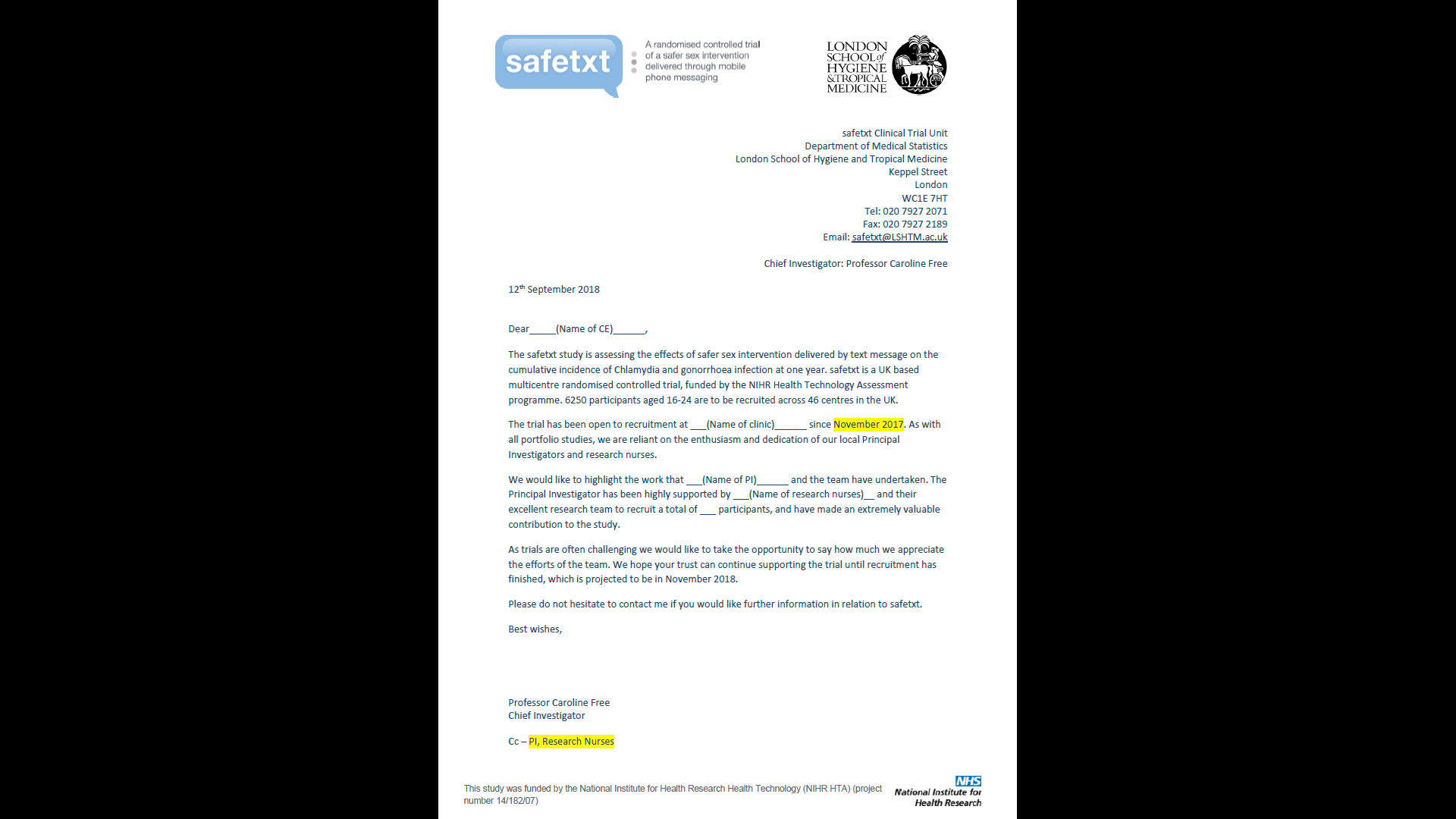

Supplement: sj-docx-6-ctj-10.1177_17407745221078882 – Supplemental material for A dynamic and collaborative approach to trial recruitment in safetxt, a UK sexual health randomised controlled trial [file sj-docx-6-ctj-10.1177_17407745221078882.docx]

## Appendix 7. Example of a recruitment certificate.


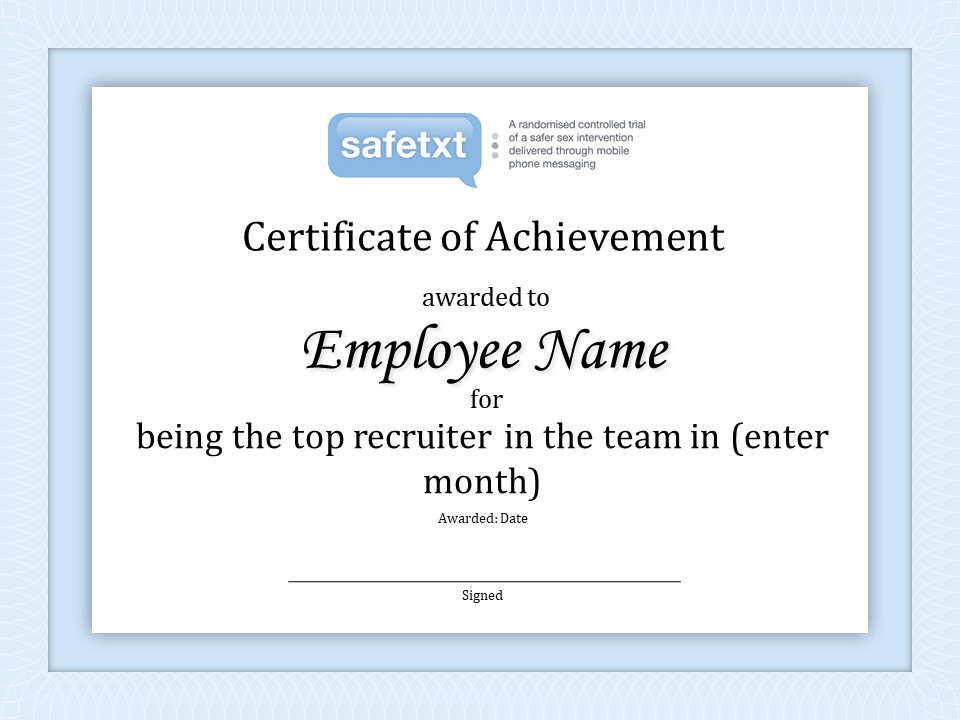

Supplement: sj-docx-7-ctj-10.1177_17407745221078882 – Supplemental material for A dynamic and collaborative approach to trial recruitment in safetxt, a UK sexual health randomised controlled trial [file sj-docx-7-ctj-10.1177_17407745221078882.docx]
