# Supplementary material for: A dynamic and collaborative approach to trial recruitment in safetxt, a UK sexual health randomised controlled trial
Source: Clin Trials. 2022 Mar 5;19(3):251–8. doi: 10.1177/17407745221078882 (PMC9203664; doi:10.1177/17407745221078882)
Supplement: sj-docx-3-ctj-10.1177_17407745221078882 – Supplemental material for A dynamic and collaborative approach to trial recruitment in safetxt, a UK sexual health randomised controlled trial [file sj-docx-3-ctj-10.1177_17407745221078882.docx]

## Appendix 3. Pie chart showing different reasons for declinations, and the percentage of participants (out of 834) who gave each reason. Data taken from screening logs.
