# Supplementary material for: A dynamic and collaborative approach to trial recruitment in safetxt, a UK sexual health randomised controlled trial
Source: Clin Trials. 2022 Mar 5;19(3):251–8. doi: 10.1177/17407745221078882 (PMC9203664; doi:10.1177/17407745221078882)

## Appendix 8. Newsletters.

*
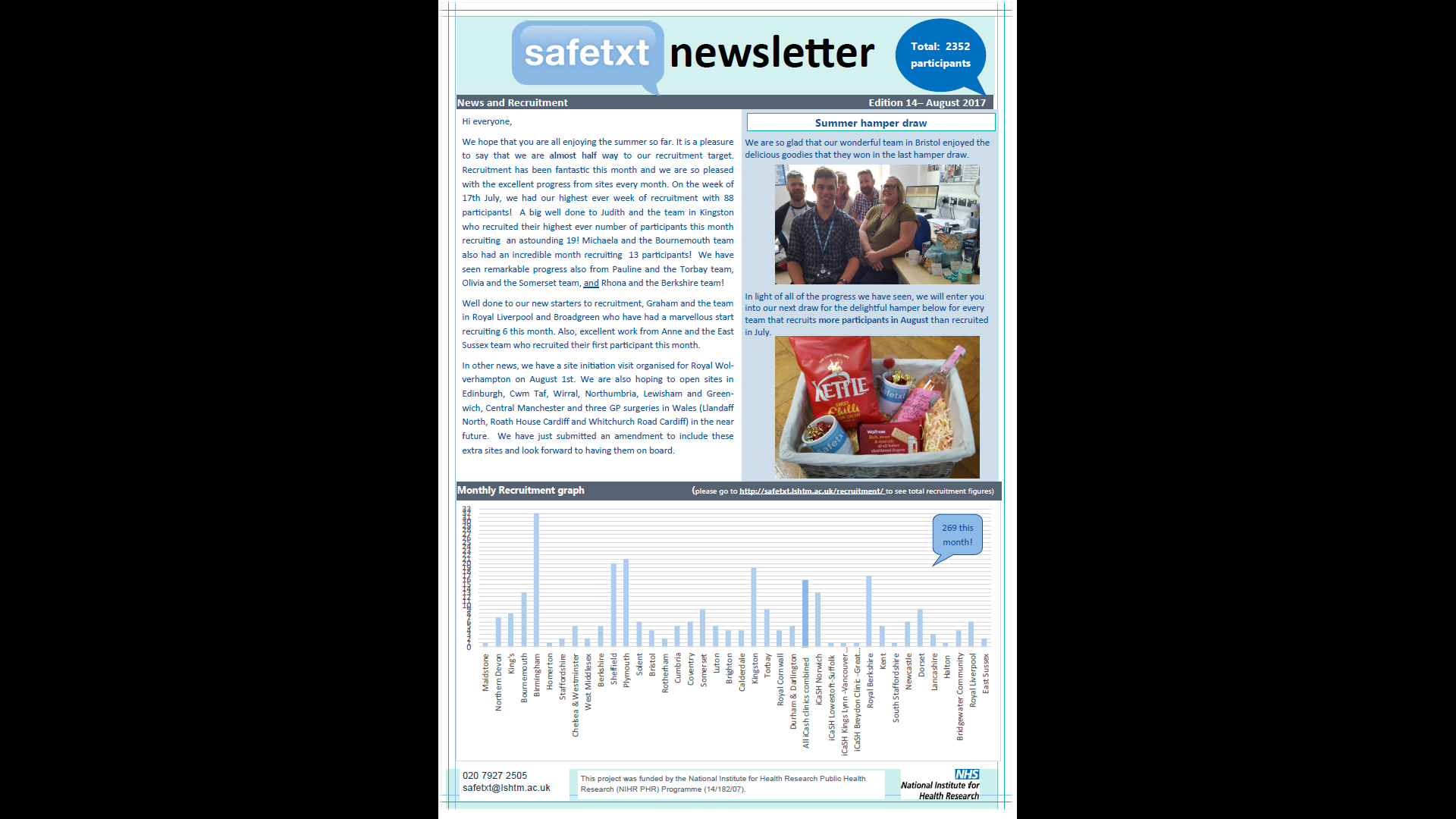
Newsletter 1 - Front*

*Newsletter 1 - Back*

##
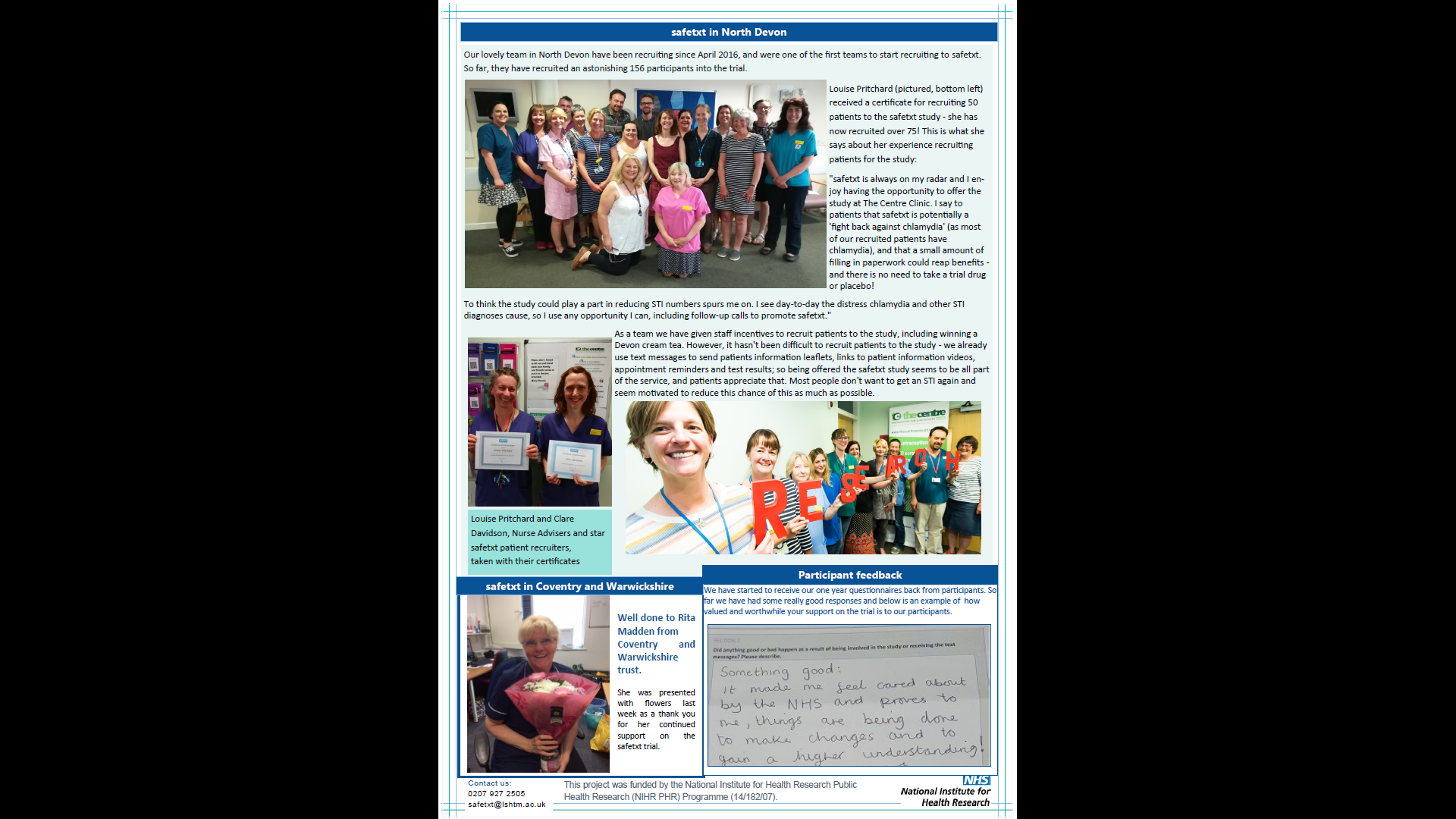


*Newsletter 2 - Front*


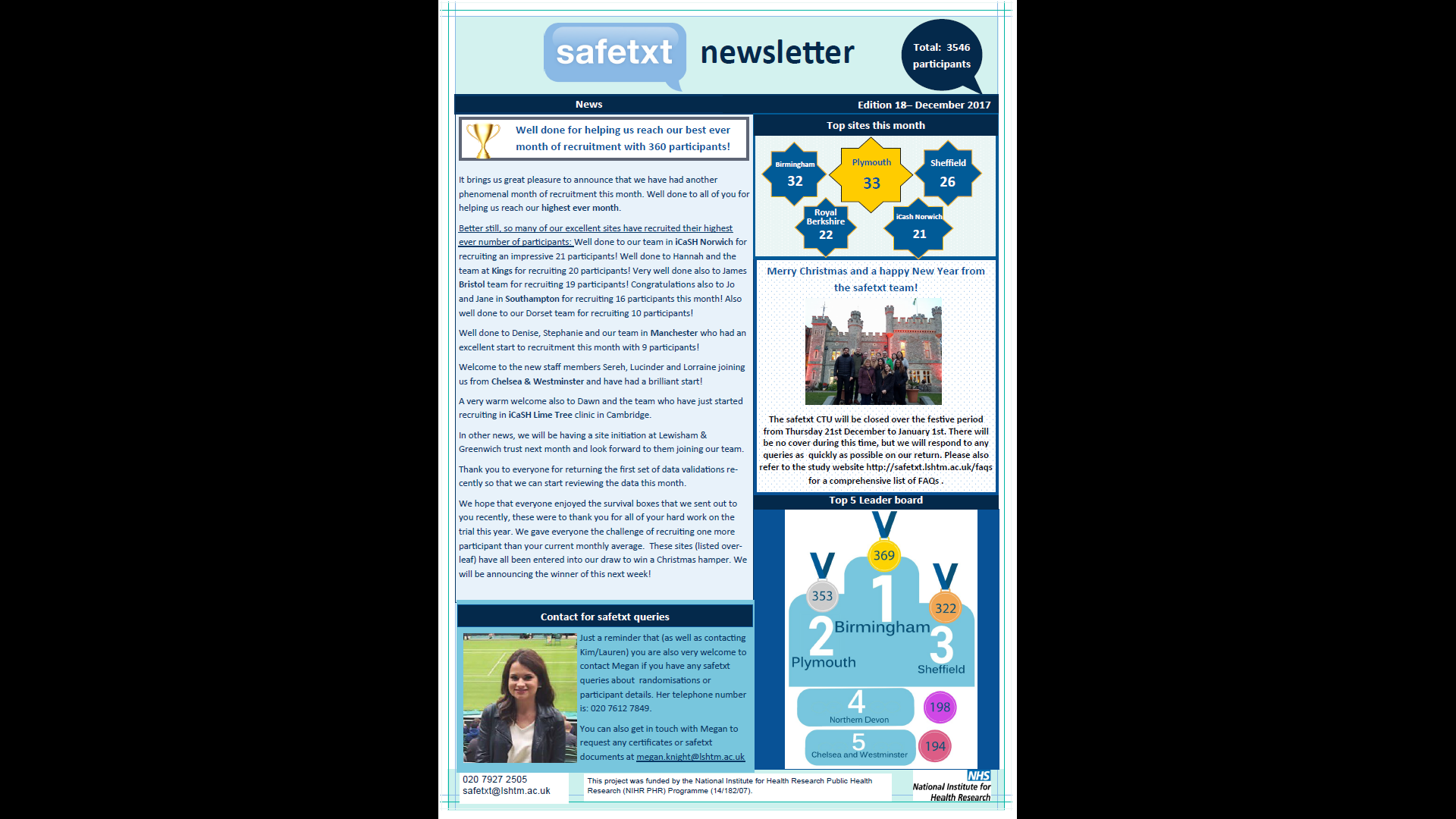


*Newsletter 2 - Back*


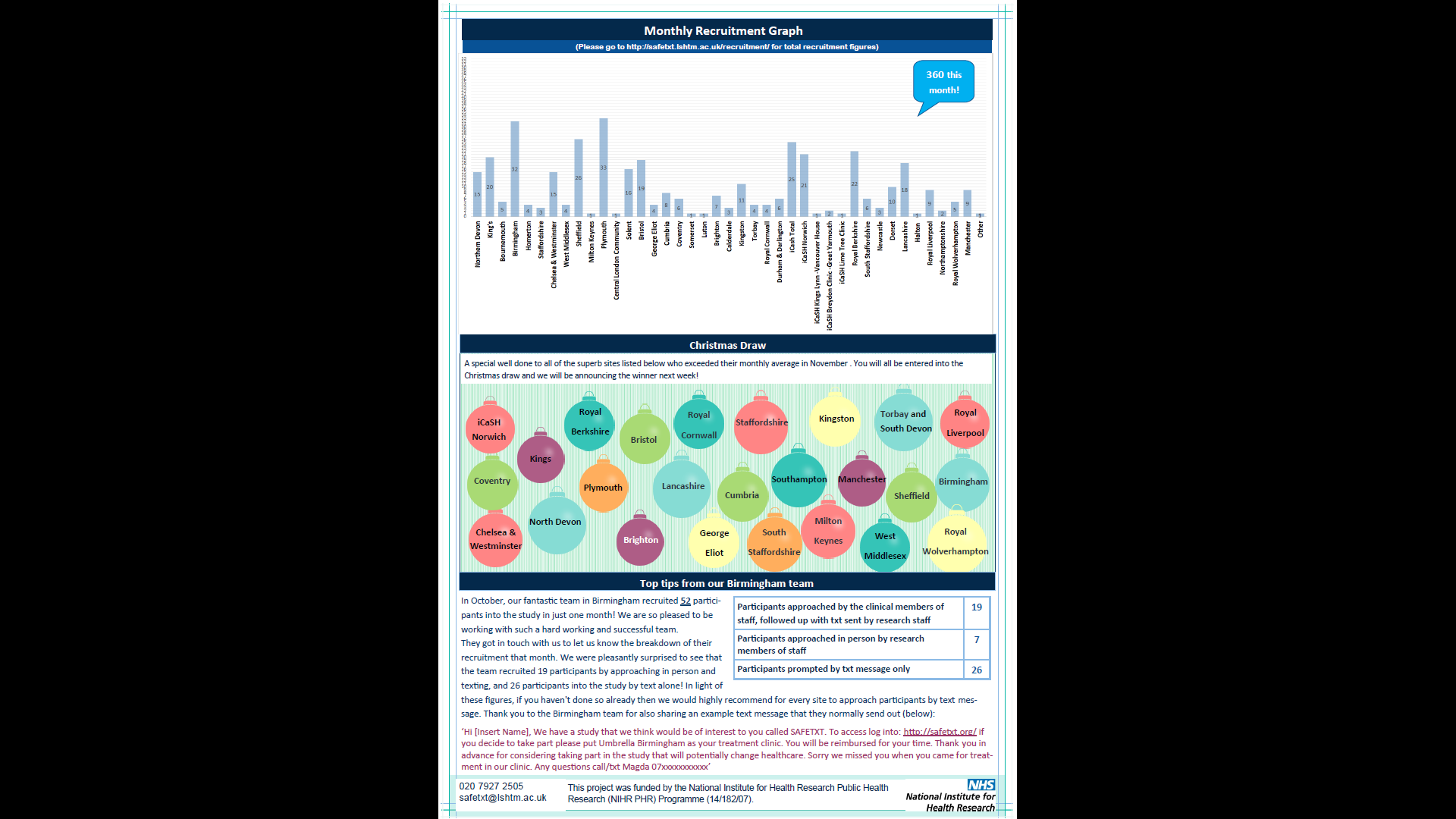


*Newsletter 3 - Front*


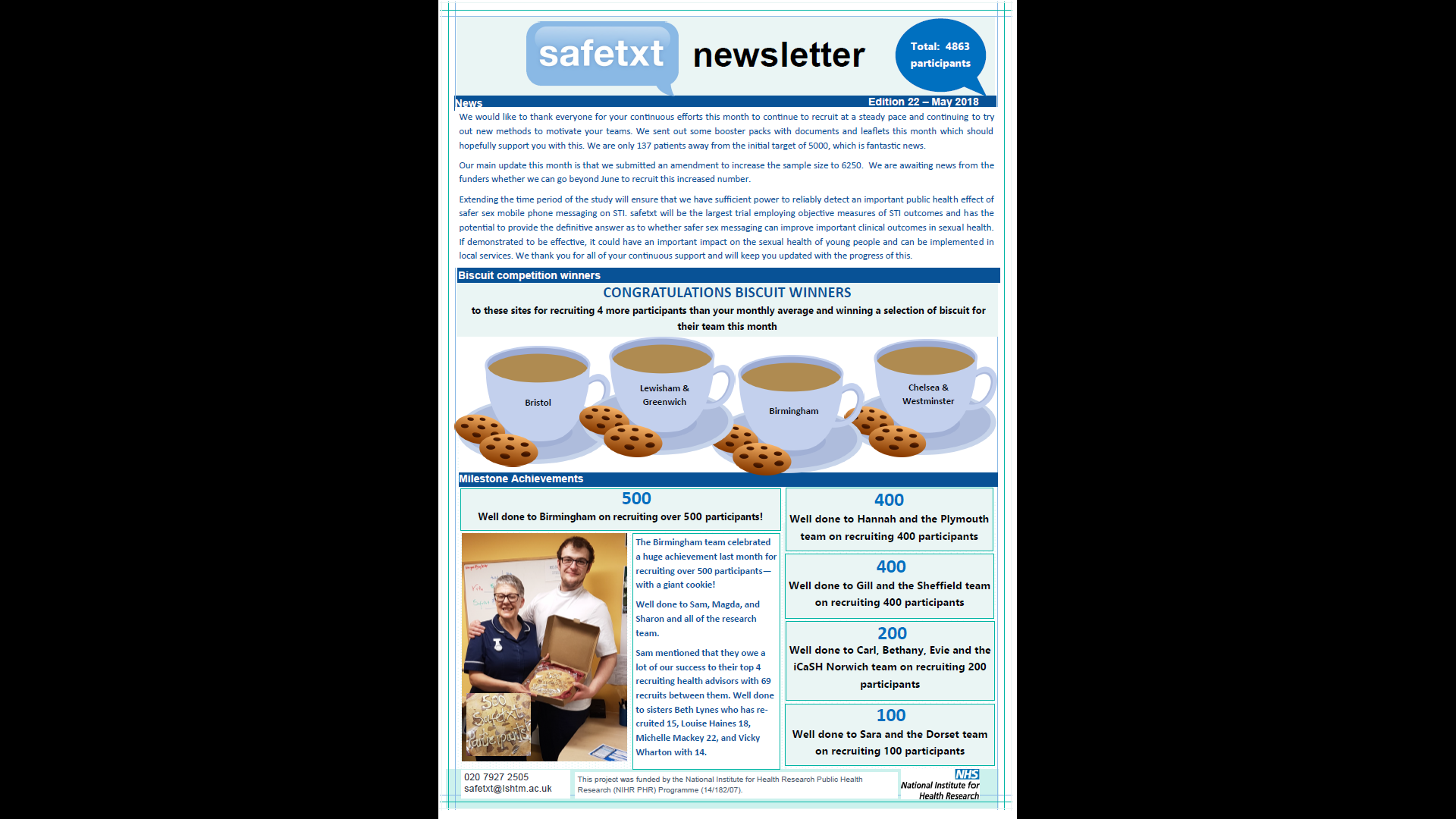


*Newsletter 3 - Back*


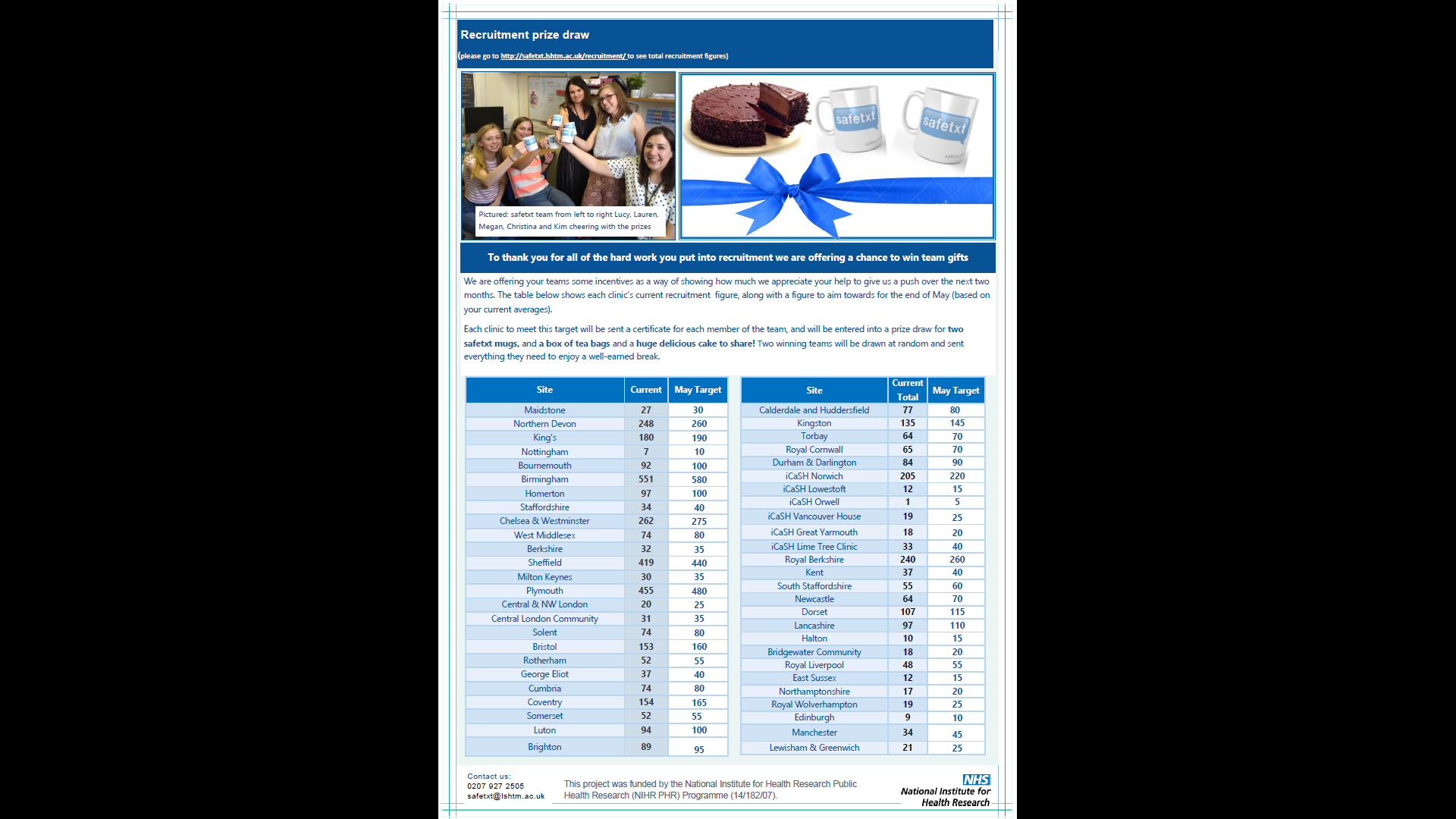

Supplement: sj-docx-8-ctj-10.1177_17407745221078882 – Supplemental material for A dynamic and collaborative approach to trial recruitment in safetxt, a UK sexual health randomised controlled trial [file sj-docx-8-ctj-10.1177_17407745221078882.docx]
